# Use of Ultrasonic Device in Cervical and Thoracic Laminectomy: a Retrospective Comparative Study and Technical Note

**DOI:** 10.1038/s41598-018-22454-y

**Published:** 2018-03-05

**Authors:** Yu Chen, Zhengqi Chang, Xiuchun Yu, Ruoxian Song, Weimin Huang

**Affiliations:** General Hospital of Jinan Military Commanding Region, Jinan, Shandong province P.R. China

## Abstract

Multilevel severe compressive myelopathy is a challenging disorder for the surgeons, the aim of this study is to assess the efficacy and safety of a newly designed ultrasonic burr as an assistant tool to the ultrasonic scalpel in laminectomy for this disease. This is a retrospective comparative study, the included subjects were patients who received cervical and thoracic laminectomy using ultrasonic device (LUD, n = 9, 10 surgeries) and controls with the high-speed burr (LHB, n = 16). Fifteen patients (60.0%) showed severe cord occupancy and the average number of laminae operated was 3.5. Ultrasonic devices caused less blood loss (P = 0.02) and quicker operative time per level (P < 0.001) than LHB, and was associated with more operated laminae (P = 0.04). Preoperative JOA scores (P = 0.51), improvement rate (P = 0.47), and dural injury (P = 0.51) were not related to LUD. Our experience indicates ultrasonic devices are safe and effective for laminectomy treating multilevel and severe compressive myelopathy, the instrument could be used with ease especially for cases with ossified posterior longitudinal ligament and ossification of the ligamentum flavum, proper utility of the instrument is crucial to prevent complications.

## Introduction

Compressive myelopathy is a progressively disabling disorder, the main etiology includes ossification of the posterior longitudinal ligament (OPLL), ossified ligamentum flavum (OLF) and spinal degenerative disorders^1^. Severe compression of the cord (spinal cord occupancy rate ≥60%) complicated with symptom exacerbation usually requires surgical treatment because conservative therapy is of no benefit^2^. Surgical procedures treating multilevel severe lesions are technically demanding and risky, especially when the patient presents with OPLL (cervical, 3.2%; thoracic, 0.8%) with or without OLF^1^. Reports in previous literature have employed several surgical approaches for compressive myelopathy including posterior circumferential decompression^2,3^, anterior decompression^4^, posterior laminectomy or laminoplasty^1,5,6^, and anterior combined with posterior decompression^1,7^. Each surgical protocol has its advantages and disadvantages, anterior procedures and circumferential decompression yield relatively satisfactory neurologic improvement rate, however, these surgical methods are technically demanding and accompanies with high incidence of complications including dural tear (40–63%), postoperative neurologic deterioration (29–33%) and deep infection (10%)^8–10^. Posterior laminectomy and laminoplasty provide indirect decompression by posterior shift of the spinal cord, these procedures are especially applicable for multilevel and severe myelopathy for which direct decompression is risky, however, these posterior decompressive techniques are associated with inferior neurologic improvement and notable complications^11^. Additionally, concomitant OPLL and OLF predispose to severe myelopathy and present great challenges to the surgeons^6^, therefore it’s imperative to establish surgical procedures with favorable efficacy and safety for this disorder.

The high morbidity correlated with treatment of multilevel and severe compressive myelopathy is due to increased technical difficulty and inherent shortages of traditional surgical instrumentation. High-speed burrs are widely used in spine surgeries for osteotomy, however, the heat generated by the tip of the device may cause damage to neighboring neural tissue and result in osteonecrosis^12^. For subjects with severe spinal stenosis where the epidural space is extremely narrow, it’s almost impossible to use a Kerrison rongeur for decompression, other tools may also easily cause mechanical damage to the dura, spinal cord, nerve roots, and vessels. In addition, a downward pressure during surgical manipulation is usually inevitable and might aggravate the myelopathy^13^.

Ultrasonic device has been recently used as an assistant tool for osteotomy in spine surgeries^13–20^. Its tip vibrates at an ultrasonic frequency and selectively cut the mineralized structures such as bone, while the underlying soft tissue (e.g. dura, cord, nerve roots, and vessels) is spared because it could resolve the energy by greater tissue compliance^15,19,20^. Previous articles have employed several types of device tips including the scalpel^14,21^, curette^17,18^, and microhook shaver^22^, preliminary studies certified their efficacy in various spine surgeries^14–16^. Nevertheless, limited articles focused on the application of ultrasonic devices in multilevel severe compressive myelopathy, which we believe is an ideal indication for this tool. In addition, all the reported device tips are designed with a one-side cutting surface for osteotomy. Herein we use a newly designed ultrasonic burr as an assistant tool to the scalpel tip for posterior laminectomy with instrumented fusion, and analyze the technical aspects of this device.

## Materials and Methods

### Patient population

This is a retrospective case-control study. From January 2015 through June 2016, two experienced surgeons performed cervical and thoracic laminectomy using an ultrasonic device (LUD) in 9 subjects (10 surgeries), the details of these patients are listed in Table 1. There were 5 female patients (55.6%) and 4 male subjects (54.4%) with an average age of 57.3 ± 11.6 years (range, 45–76 years). We also selected 46 cases undergoing posterior laminectomy assisted by a high-speed burr (LHB) with the age and sex comparable to those receiving LUD, and randomly chose 16 controls from them, there were 9 men (56.2%) and 7 women (43.8%) with an average age of 57.8 ± 8.7 years (range, 41–70 years). The baseline characteristics of the total subjects are presented in Table 2. Of all the cases, the etiology included concomitant OPLL and OLF in 3 subjects (thoracic + cervical, thoracic + lumbar, and whole spine in 1 respectively, 12.0%), cervical OPLL in 4 (16.0%), OLF in 5 (thoracic in 3, cervical in 1, and thoracolumbar in 1, 20.0%), cervical spondylotic myelopathy in 5 (20.0%), thoracic spinal stenosis in 5 (20.0%), and hyperextensive injury of cervical spinal cord in 3 (12.0%). The current study was approved by the Ethics Committee of General Hospital of Jinan Military Commanding Region and every patient provided a written informed consent. The methods described were carried out in accordance with relevant guidelines and regulations. The authors declare that this was not a study commissioned or funded by the manufacturer of the device.

**Table 1:** Baseline characteristics of the patients undergoing ultrasonic device assisted laminectomy.

No. patients	Sex	Age (y)	Body mass index (Kg/m^2^)	Etiology	Urinary dysfunction	Follow up (m)	Preop. JOA	Improvement rate (%)
1	Female	49	23.4	TL-OPLL+OLF^a^	Yes	30	4	46.2
2	Female	46	19.8	C-OPLL^b^	N	25	8	33.3
3	Female	45	26.2	CTL-OPLL+OLF^c^	Yes	12	5	41.7
4	Male	73	22.3	C-OPLL	N	25	10	42.9
5	Male	60	21.2	T-OLF^d^	N	18	11	33.3
6	Female	58	22.9	TDH^e^	N	23	11	50.0
7	Female	76	20.2	CSM^f^	N	22	5	66.7
8	Male	49	24.5	TL-OLF^g^	N	13	11	66.7
9	Male	68	23.5	T-OLF	N	12	9	50.0

^a^TL-OPLL+OLF denotes thoracic and lumbar ossification of the posterior longitudinal ligament (OPLL) coexisted with ossified ligamentum flavum (OLF); ^b^C-OPLL denotes cervical OPLL; ^c^CTL-OPLL + OLF denotes cervical, thoracic and lumbar OPLL complicated with OLF; ^d^T-OLF denotes thoracic OLF; ^e^TDH denotes thoracic disc herniation; ^f^CSM denotes cervical spondylotic myelopathy; ^g^TL-OLF denotes thoracolumbar OLF.

**Table 2:** Comparison of the ultrasonic device and the high-speed burr.

Parameter	Ultrasonic device	High-speed burr	P value
Age (y)	57.3 ± 11.6	57.8 ± 8.7	0.89
Male, n(%)	4(44.4)	9(56.2)	0.69
Average CR^a^ (%)	63.0	57.2	0.30
Blood loss (ml)	435.0 ± 159.9	703.1 ± 295.8	0.02
Time per level (min)	3.7 ± 0.7	8.0 ± 1.2	<0.001
Blood transfusion, n(%)	3(30.0)	8(50.0)	0.43
Dural tear, n(%)	N	2(12.5)	0.51
No. of lamina operated	4	3	0.04
Compression rate (%)	63.0 ± 10.2	57.2 ± 15.5	0.30
Preoperative JOA score	8.3 ± 2.7	9.3 ± 3.9	0.51
Improvement rate (%)	45.6 ± 13.6	49.7 ± 14.2	0.47

^a^CR denotes compression rate of the spinal canal.

### Assessment of the lesions responsible for myelopathy

We determined the diagnosis of myelopathy by interpreting the physical examination and magnetic resonance imaging presentations. On an axial view of computed tomography, spinal cord compression rate (CR) was defined as CR = longest sagittal extension of the lesion/sagittal diameter of the spinal canal × 100%^23^. Among all the cases, there were 15 subjects (60.0%) showing severe compression of spinal cord with CR ≥ 60%, the average CR was 59.0 ± 13.8% (range, 29–83%), of cases with OPLL, OLF and both (12 subjects), 9 cases (75.0%) presented with severe CR of 71.0 ± 7.0% (range, 62–83%). Classification of OPLL is determined in accordance with the system developed by Investigative Committee on the OPLL of the Japanese Ministry of Public Health and Welfare^24^, it categorizes the disorder into four types based on the lateral view of radiography: continuous, segmental, mixed and localized, in this series, six out of seven OPLL cases (85.7%) presented with continuous lesions, while one subject (14.3%) exhibited mixed OPLL. Modified Japanese Orthopedic Association (JOA) scoring system was used to evaluate the neurologic improvement^25^. Improvement rate (IR) was calculated as IR = (postoperative JOA score-preoperative JOA score)/(17-preoperative JOA score) × 100%. Excellent recovery was defined as an IR of 75% or greater, good as 75% > IR ≥ 50%, fair as 50% > IR ≥ 25%, and poor as IR < 25%^25,26^.

### Surgical techniques

A variety of surgical techniques are applicable for treating compressive myelopathy^1–5^, we selected posterior laminectomy with instrumented fusion in the current cases for following reasons: (1) most of patients in the present study were with severe impingement of spinal cord by multilevel lesions, in this setting, the epidural space is narrow or even missing, anterior decompression and posterior circumferential procedures predispose to iatrogenic injuries and accompany with various complications such as dural tear^13,16^; (2) posterior laminectomy is technically easier and safer in comparison with direct decompression of the cord, although several authors argued that posterior decompression provides inferior neurologic recovery with an IR less than 50%^11–13^, surgical outcomes reported by previous literature suggested good neurologic improvement is possible after laminectomy with instrumented fusion despite residual anterior impingement of the cord^11^. (3) most OPLL responsible for symptomology in the present study were categorized as continuous type, there were no beak-type OPLL which is associated with poor outcomes after laminectomy^27^.

**Figure 1:**
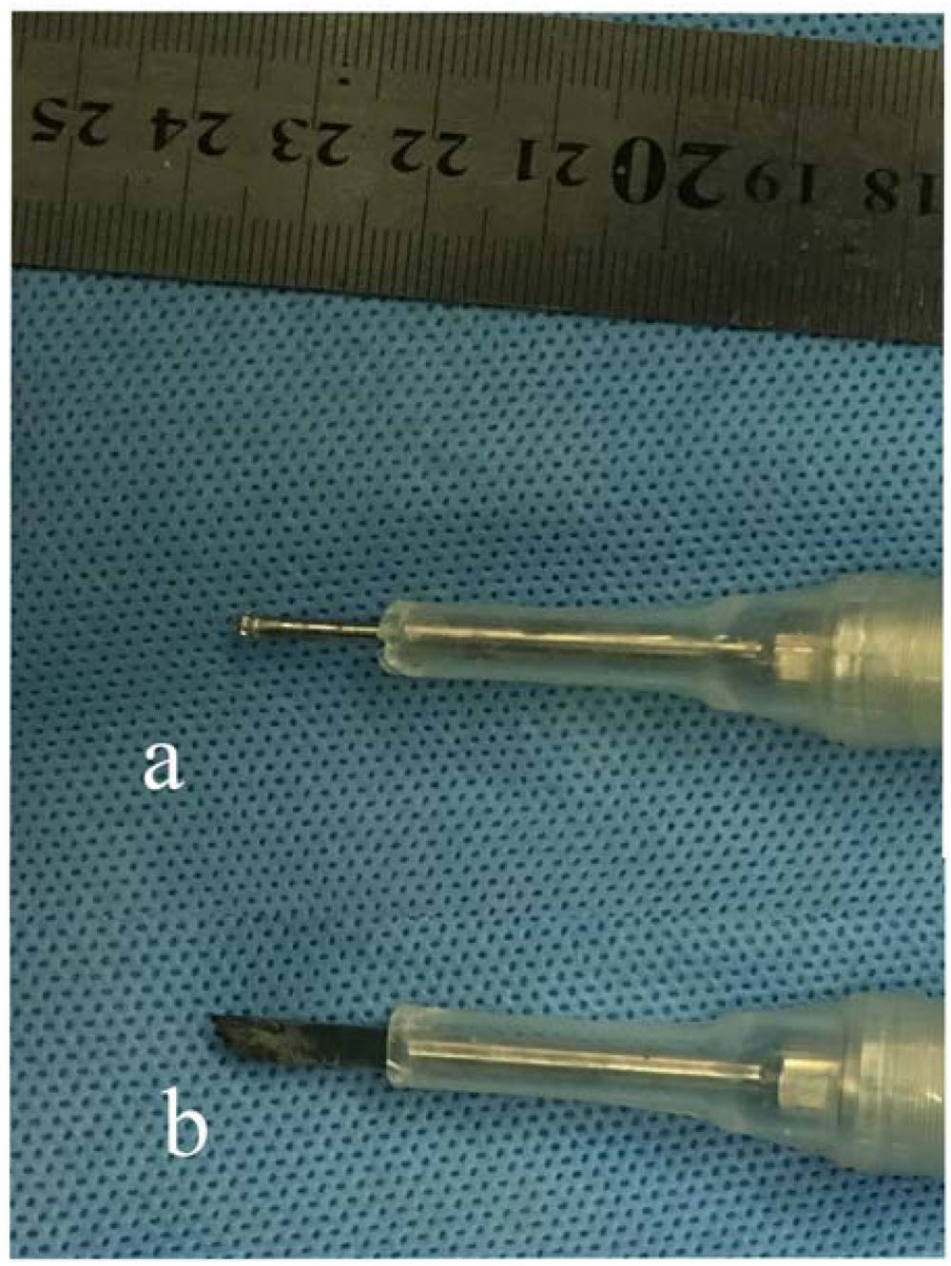
Ultrasonic device tips used for laminectomy. Figure 1a demonstrates a newly designed burr tip used to widen the cutting corridor and decrease the thickness of lamnae to be addressed, it’s especially feasible for cases with ossified ligamentum flavum; b shows a thin scalpel tip designed for osteotomy.

A standard posterior midline approach was used to expose the spinous processes, laminae, and facet joints. Instrumentation with lateral mass screws (cervical) and pedicle screws (thoracic and lumbar) were performed, and a rod is fixed on one side. The supraspinous and interspinous ligament were cut at the cranial and caudal edges of the planned decompression field respectively. We used an ultrasonic bone cutting device (XD860A, SMTP Technology Co., Ltd, CHN) to conduct bilateral linear osteotomies for laminectomy (for the control group we used the high-speed drill and Kerrison rongeur for osteotomy), the device is equipped with a safety stop at the blade end, its tip oscillates at a frequency of 39000/s with the maximum amplitude no more than 0.12 mm, this tool could selectively cut the mineralized structures while leaving the underneath critical tissue atraumatic, additionally a downward pressure on the spinal cord is avoidable with limited thermal effect, we believe these strengths make it applicable for severe compression of cord where epidural space is narrow or missing. In cervical surgeries, the osteotomy grooves were located at the conjunction of the lamina and the lateral mass, while thoracic laminectomy usually entails cutting at a distance of approximately 1 cm to the midline, it’s noteworthy that the cutting corridor should not be placed too laterally because manipulation with the hand piece may interfere with the screws, which may cause damage to the device tip. A thin scalpel (15 mm in length, Fig. 1b) was used for osteotomy, continuous irrigation with physiologic saline helped to minimize the local thermal effect. Among cases with OLF, the thickness of lamina to be cut is large, it’s usually difficult to perform laminectomy solely with an ultrasonic scalpel due to limited length of the tip and restricted visibility due to narrow cutting corridor, in this setting we used an ultrasonic burr (1.5 mm in diameter, Fig. 1a) to widen the cutting groove and decrease the thickness of residual bone to be addressed by the scalpel, the remaining lamella could also be removed by a Kerrison rongeur. After removal of the laminae, we conducted fixation with a rod on the lateral side, and grafted the harvested bone from the extirpated laminae onto the facet joints and between the transverse processes.

### Statistical analysis

We used SPSS 13.0 software (SPSS Inc., Chicago, IL, USA) to analyze the clinical data, we applied Fishers’exact test for evaluating the categorical data, and analysis of variance was used for continuous data, the average values are presented as mean ± standard deviation, it was significant when the P value was less than 0.05.

## Results

### Operative data

The operative data of LUD is shown in Table 3. Table 2 shows the comparison of LUD and LHB, there were more operated laminae in LUD than LHB (P = 0.04), LUD causes less blood loss (P = 0.02) and quicker operative time per level (P < 0.001), the blood transfusion rate (P = 0.43) and cord compression rate (P = 0.30) were not related to the use of ultrasonic devices.

**Table 3:** Operative data of the patients.

No. patients	Operated levels	Types of OPLL	Blood loss (ml)	Blood transfusion (ml)	Operative time (min)	Hospital stay (d)	Complications
1	1^st^,T2–9^a^ 2^nd^,T10-L2	Mixed	1^st^, 500 2^nd^, 600	1^st^, 200 2^nd^, 400	1^st^, 207 2^nd^, 226	1^st^, 17 2^nd^, 32	1^st^, N 2^nd^, delayed wound healing
2	C3–6	Continuous	300	N	117	14	N
3	T6–11	Continuous	800	400	325	16	N
4	C3–6	Continuous	400	N	202	13	N
5	T10-L1	N	400	N	165	10	N
6	T10-L1	N	300	N	160	15	N
7	C3–6	N	300	N	103	13	N
8	T12/L1	N	350	N	156	13	N
9	T6–8	N	400	N	160	15	N

^a^1^st^ and 2^nd^ denote the first and second stage operation.

### Postoperative outcomes

The patients were followed up with an average duration of 23 ± 6 months (range, 12–31 months), no subject was lost to follow-up. Preoperative JOA score was 8.9 ± 3.5 (range, 4–15), at the final follow-up, the average score measured 12.7 ± 2.3 (range, 7–16), the mean improvement rate was 48.1% ± 13.9% (range, 33.3–72.7%). According to our criteria, 13 cases (52.0%) experienced good neurologic recovery while 12 patients (48.0%) exhibited fair recovery. Preoperative JOA scores (P = 0.51) and postoperative improvement rate (P = 0.47) were similar between the two surgical procedures.

### Complications and treatment

No cases having LUD experienced dural injuries, while 2 subjects (12.5%) had dural tears after LHB, it was successfully treated by intraoperative closure using 3–0 nylon sutures and coverage with fascia patch, the incidence of dural injury (P = 0.51) was not associated with the use of ultrasonic devices. There was no transient neurologic deterioration, one patient with thoracic myelopathy reported intercostal pain postoperatively. We found no deep infection, 1 patient experienced delayed wound healing after a second stage operation, which was successfully treated by frequent dressing change until hospital discharge. One subject presenting with concomitant OPLL and OLF involving both thoracic and lumbar vertebrae underwent staged surgery, after initial thoracic spinal decompression she experienced fair neurologic improvement measured by IR, at 12 months postoperatively weakness complicated with sensory deficit was reported, therefore she received secondary decompression in the remaining thoracic and lumbar levels.

## Discussion

**Figure 2:**
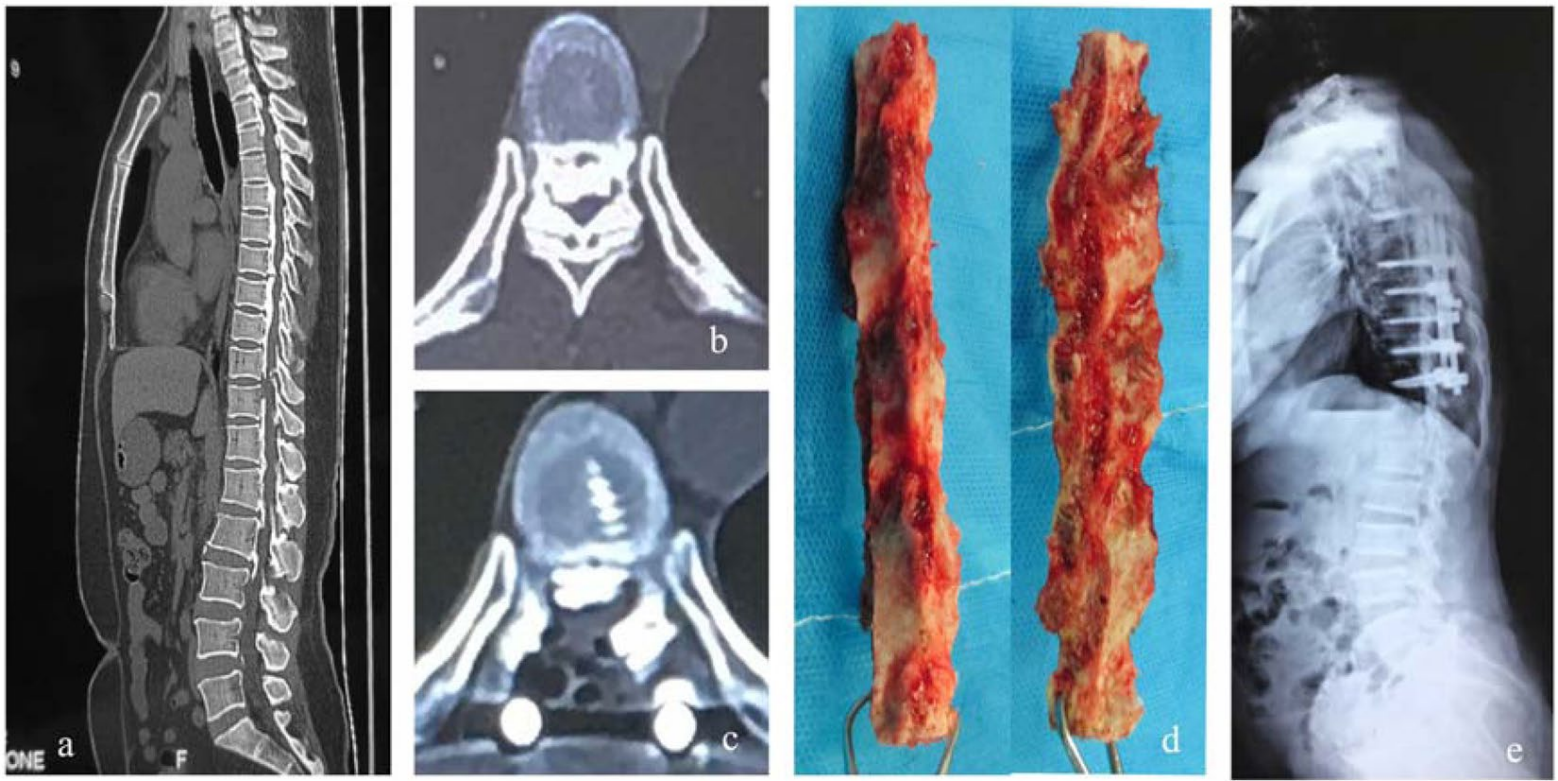
Utility of ultrasonic device in thoracic ossified posterior longitudinal ligament (OPLL) complicated with ossification of the ligamentum flavum (OLF) 45-year old female patient presented with weakness and numbness of the lower extremities, she also had urinary dysfunction and was unable to ambulate (JOA, 5). Figure 2a: computed tomography shows continuous OPLL ranging from cervical to lumbar levels; Fig. 2b and c demonstrate severe impingement of cord by concomitant OPLL and OLF and posterior shift of the spinal cord after operation; Fig. 2d shows the resected laminae by posterior laminectomy with instrumented fusion (T6–11); Fig. 2e demonstrates the postoperative radiograph of the spine. She experienced improvement rate of 41.7% without complications 12 months postoperatively.

**Figure 3:**
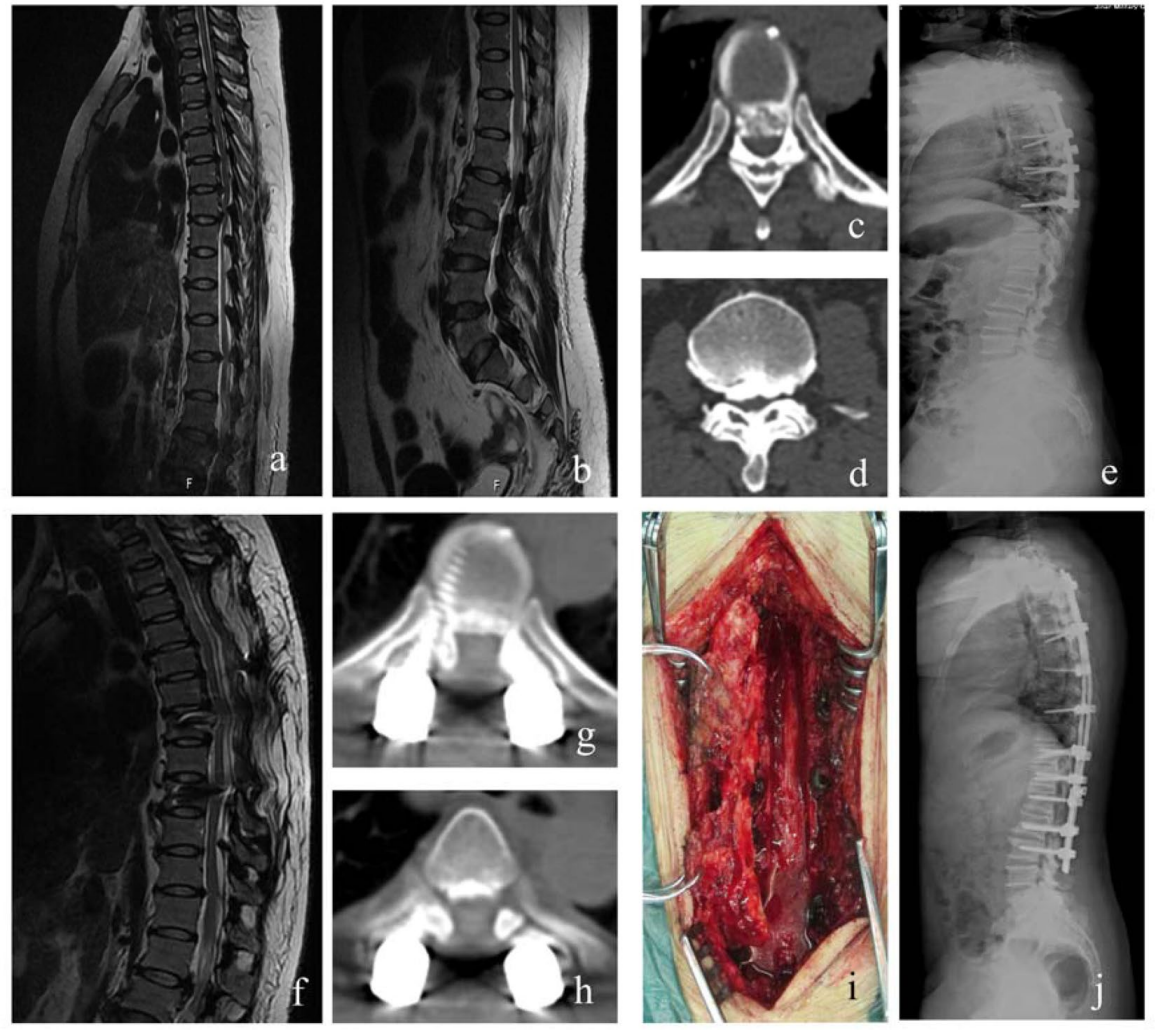
Coexisting ossified posterior longitudinal ligament (OPLL) and ossification of the ligamentum flavum (OLF) involving thoracic and lumbar levels treated with staged laminectomy using the ultrasonic device A 49-year old female patient exhibited paraplegia (JOA, 4), magnetic resonance images (MRI) showed concomitant OPLL and OLF involving T2–9 and T10-L2 levels (**a**,**b**); computed tomography (CT) indicated severe spinal canal occupancy at thoracic (**c**) and lumbar (**d**) levels; the patient underwent thoracic laminectomy first (**e)**, 12 months after operation, ideal thoracic decompression was demonstrated by MRI (**f**) and CT (**g**,**h**); she had second operation for OPLL and OLF in T10-L2 levels at an interval of 12 months (**i**,**j**). She had a 46.2% improvement rate 30 months postoperatively.

Ultrasonic oscillation for osteotomy was reported decades ago, however, the instruments were used in spine surgeries only in the last several years^17^ (Table 4). Previous articles claimed the advantages of ultrasonic device include decreased risk of mechanical injury, limited heat production, reduction in osseous bleeding which allows for visibility in the surgical field, and shortened time for laminar removal^17–22^, however, restricted literature focused on its application in laminectomy for severe impingement of the cord. We believe this instrument provides ideal features for this disorder, the relevant strengths include: (1) because the device tip operates with microvibration that is imperceptible, it allows for osteotomy with satisfactory precision and ease of control; (2) among cases with significant cord occupancy, the epidural space is too narrow for decompression using traditional instrumentation, in cases with concomitant OPLL and OLF, utility of a Rongeur is impossible, and a high-speed burr would be risky, in contrast, ultrasonic devices could be used without any downward pressure on the cord, it also minimizes the risk of mechanical and thermal injury to the neighboring critical tissue; (3) Onen, MR *et al*.^16^ reported the time for decompressing each level was 2.2 minutes using an ultrasonic device, in the current cohort, we found similar outcomes, and LUD was quicker than the high-speed burr, due to the favorable visibility and ideal time for removal of each lamina, an ultrasonic device allows the surgeons to perform multilevel decompression of cord, which is frequently necessary in myelopathy resulting from coexisting OPLL and OLF (Figs 2 and 3). For cases with concomitant OPLL and OLF, posterior laminectomy usually needs to cut the thickened laminae, because the ultrasonic bone scalpel creates very narrow bone cavities in a linear fashion, and the length of the tip is restricted by the safety stop at the end of the blade, these factors increased the difficulty for osteotomy on laminae of significant thickness, therefore we applied a newly designed burr shape tip to widen the cutting corridor and decrease the thickness of the lamina to be cut by the scalpel, our experiences indicate concomitant application of these two tips provided favorable efficacy and safety in laminectomy for severe compressive myelopathy, especially for coexisting OPLL and OLF.

**Table 4:** Key literature regarding the ultrasonic device assisted spine surgery.

No.	References	Sample size (n) & control(Yes/N)	Basic data	Operative techniques	Levels of the spine	Mean levels operated	Complications n (%)	Mean blood loss (ml)	Mean operation time (min)
1	Hu, X.B., *et al*.^14^	128,N	Female, 73 Male, 55 Mean age, 58 y Follow up, N	Facetectomy, laminotomy, laminectomy, corpectomy, osteotomy	All levels	5	Dural laceration, 2 (1.6)	425	258
2	Morimoto, D., *et al*.^15^	26,N	Female, 11 Male, 15 Mean age, 60 y Follow up, 31 m	Fenestration	Lumbar	N	Recurrence of L5 radiculopathy, 1(3.8)	N	N
3	Onen, M.R., *et al*.^16^	23,Yes (recovery rate comparable between two groups)	Female, 5Male, 18 Mean age, 61 y Follow up, N	Laminectomy	Cervical	3	C5 radiculopathy, 1(4.3)^a^	180^b^	2.2/level^c^
4	Kim, K., *et al*.^17^	546,N	Female, 192 Male, 354 Mean age, 55 y Follow up, N	Hemilaminecto my,laminoplasty, foraminotomy, lateral recess exposure	All levels	N	Dural laceration, 5(0.9), neurological aggravation, 1(0.2)	N	N
5	Nakagawa H., *et al*.^18^	70,N	Female, 40 Male, 30 Mean age, 61 y Follow up, N	Microsurgical decompression	All levels	N	N	N	N
6	Kunpeng, Li., *et al*.^20^	42,Yes (neurological recovery comparable between 2 groups)	Female, 11 Male, 31 Mean age, 57 y Follow up, N	Laminectomy	Cervical	≥ 3	C5 paralysis, 2(9.5) Dural laceration, 1 (4.8)^d^	273^e^	138 ^f^
7	Al-Mahfoudh, R., *et al*.^21^	62,N	N	Laminotomy, corpectomies	All levels	N	Dural laceration, 4 (6.5) Wound infection, 2(3.2) Neurological aggravation, 2 (3.2) C5 root lesion, 1 (1.6)	N	N
8	Hazer, D.B., *et al*.^22^	307,N	Female, 182 Male, 125 Mean age, N Follow up, N	Laminectomy, foraminotomy, corpectomy, resection of calcified disc and endplate	All levels	N	Dural laceration, 5 (1.6)	N	Lumbar,73–196 Thoracic,169–213 Cervical, 107–154

^a^Denotes ultrasonic devices resulted in less dural tears than high speed drills (0 vs. 13.3%, P < 0.05) while occurrence of C5 paralysis was similar; ^b^denotes ultrasonic devices caused less bleeding than high speed drills (180 vs. 380 ml, P < 0.05); ^c^denotes ultrasonic devices resulted in quicker operative time for per lamina than high speed drills (2.2 vs. 7.9 min, P < 0.05); ^d^denotes ultrasonic devices were associated with similar C5 paralysis and dural tear with high speed drills (P > 0.99); ^e^denotes ultrasonic devices related to less bleeding (273 vs. 357 ml, P < 0.05); ^f^denotes total operative time was coherent between ultrasonic devices (138 min) and high speed drills (126 min, P > 0.05).

Compressive myelopathy sometimes involve discontinuous levels including cervical, thoracic and lumbar spine, Kawaguchi Y *et al*.^28^ reported 53.4% cases with cervical OPLL had concomitant thoracic or lumbar spinal lesions, the authors recommended computed tomography analysis of the whole spine for early detection of additional sites of disorder^9^. For subjects presenting with myelopathy caused by multilevel discontinuous stenosis, staged surgical strategy is preferable, because a single-stage protocol may be too invasive. Li WJ *et al*.^26^ performed staged posterior laminectomy and circumferential decompression for cases showing multilevel thoracic OLF with or without lumbar spinal stenosis, surgical sequences were determined according to the predominant lesions responsible for the symptomology, the authors reported satisfactory efficacy and safety of this surgical protocol yet with 54.5% incidence of cerebrospinal fluid leakage after the second stage operation. In the current series, we found 1 subject (12.5%) presented with coexisting OPLL and OLF ranging from thoracic to lumbar spine (Fig. 3), and 1 case with discontinuous lesions involving the whole spine (Fig. 2). The former patient received staged posterior laminectomy with instrumented fusion, thoracic myelopathy resulting from upper and mid thoracic OPLL coexisted with OLF was treated first, at an interval of 12 months, a second stage operation was performed to decompress the stenosis from T10 to L2 levels. The patient experienced fair neurologic improvement without complications, however, we observed that she showed slightly inferior neurologic status before the second stage surgery, indicating the interval between the two staged operations should be shortened, our results correspond to the outcomes reported in previous literature, Li WJ *et al*.^26^ recommended an interval of no more than 6 months before the second operation or as soon as the subject’s general condition allows for a second operation. We also suggest a staged surgical decision should take into consideration the symptomology, patients’ general condition allowing for staged operation, compliance of the subject and high motivation for improved daily activities.

It’s noteworthy that ultrasonic device minimizes but not totally avoids the effect on dura and cord, in previous articles describing the use of ultrasonic tools, dural tears (0.9–6.5%) and neurological aggravation (0.2–3.2%) were reported by several authors^14,15,17,18,20–22^. Li KP *et al*.^20^ compared the safety and efficacy of ultrasonic device and high-speed drill in cervical laminoplasty, dural laceration occurred in 1 cases (4.8%) undergoing laminoplasty using piezosurgery device. Hu XB *et al*.^14^ reported 11 dural injuries (8.6%) out of 128 patients having spine surgeries, 2 cases (1.6%) were directly associated with use of an ultrasonic scalpel. Al-Mahfoudh R *et al*.^21^ found dural damage occurred in 6.5% of the subjects, and neurological deterioration happened in 3.2% of the cases. In the present cohort, we found no dural laceration and neurological aggravation among cases having LUD. Possible factors relating to ultrasonic device associated injuries to the dura and the cord include: (1) the blade is placed on one point for a long time during osteotomy, this results in excessive thermal production causing neurological injury, we conclude that the device tip may not stop on one point for more than 5 seconds; (2) the device oscillates longitudinally at ultrasonic frequency for osteotomy, therefore handling the hand piece perpendicular to the dura meter could theoretically increase the risk of dural damage, we recommend to use the hand piece more horizontally; (3) in the present series, although the power of ultrasonic device is considered to be safe (85 wattages), we still recommend it be switched to 80% energy to prevent possible iatrogenic injury; (4) additional attention should be paid to the dura preoperatively, because ossification of the dural sac may increase the risk of dural laceration caused by the ultrasonic device^11^.

Technical points about using the ultrasonic device in laminectomy include: (1) multilevel severe impingement of the cord constitutes an ideal indication for ultrasonic tools; (2) among cases with OLF that the thickness of lamina to be cut is large, usage of an ultrasonic burr helps to widen the cutting groove and decrease the thickness of residual bone to be addressed by the ultrasonic scalpel; (3) the hand piece should be used more horizontal to the dura meter; (4) the device tip may not stop on one point for more than 5 seconds; (5) reduction of the device energy is recommendable to minimize the thermal effect of the tip. (6) because metallic materials could exert destructive impact against the device tip, therefore laminectomy may be carried out after the insertion of instrumentation only on one side.

## Conclusions

Taken together, ultrasonic device shows favorable efficacy and safety in laminectomy for multilevel severe compressive myelopathy. Use of the ultrasonic burr could facilitate the scalpel tip when cutting a thickened lamina, intermittent use of the device more horizontal to the dura meter with constant irrigation helps to prevent dural injuries, reduction in the energy is recommendable during application.
